# Clinical, Cytogenetic, and Molecular Findings in Two Cases of Variant t(8;21) Acute Myeloid Leukemia (AML)

**DOI:** 10.3389/fonc.2019.01016

**Published:** 2019-10-04

**Authors:** Lindsay Wilde, Jillian Cooper, Zi-Xuan Wang, Jinglan Liu

**Affiliations:** ^1^Department of Medical Oncology, Sidney Kimmel Cancer Center, Thomas Jefferson University, Philadelphia, PA, United States; ^2^Department of Internal Medicine, Thomas Jefferson University Hospital, Philadelphia, PA, United States; ^3^Department of Pathology, Anatomy, and Cell Biology, Thomas Jefferson University Hospital, Philadelphia, PA, United States; ^4^Department of Surgery, Thomas Jefferson University Hospital, Philadelphia, PA, United States; ^5^Department of Pathology, Anatomy, and Cell Biology, Thomas Jefferson University Hospital, Philadelphia, PA, United States

**Keywords:** acute myeloid leukemia, t(8;21), cytogenetics, core binding factor, variant

## Abstract

t(8;21)(q22;q22) is present in ~5–10% of patients with *de novo* acute myeloid leukemia (AML) and is associated with a better overall prognosis. Variants of the t(8;21) have been described in the literature, however, their clinical and prognostic significance has not been well-characterized. Molecular profiling of these cases has not previously been reported but may be useful in better defining the prognosis of this subset of patients. We present two cases of variant t(8;21) AML including clinical, cytogenetic, and molecular data.

## Background

Acute myeloid leukemia (AML) with t(8;21)(q22;q22) is known as a core binding factor AML. Along with inv(16)(p13;q22)/t(16;16)(p13;q22), this cytogenetic abnormality has been shown to have a more favorable prognosis, especially when treated with high dose cytarabine based therapy (1–3). The t(8;21) is found in ~5–10% of *de novo* AML and results in the creation of the fusion gene RUNX1-RUNX1T1 (1, 4–6). In most affected individuals, the chromosomal breakpoints are located at intron 5 of the RUNX1 gene and intron 1 of the RUNX1T1 resulting in an in-frame fusion of the N-terminal 177 amino acids of RUNX1 with almost the entire RUNX1T1 protein (7). The RUNT domain from RUNX1, the four nervy homology regions (NHR), and a nuclear localization signal (NLS) from RUNX1T1 are major functional domains of the fusion protein (8, 9). By direct or indirect binding to target DNA regions, the RUNX1-RUNX1T1 regulates the expression of various groups of genes involved in multiple signaling pathways. It has been shown that the RUNX1-RUNX1T1 alone is not sufficient for leukemogenic transformation, and the number of mutations necessary for the development of AML1-ETO leukemia is still unknown (5, 6, 10). Variant translocations account for ~3–4% of leukemias with RUNX1/RUNX1T1 fusion transcripts, some of which are cytogenetically cryptic and can only be identified by molecular approaches (e.g., quantitative PCR). Clinical consequences of these variants are poorly defined (11–15). Here, we present two similar cases of variant t(8;21) identified at our institution, their molecular findings on next generation sequencing, and their clinical outcomes.

## Materials and Methods

### Conventional Cytogenetic Analysis

The bone marrow specimens were cultured in *MarrowMAX* medium (Invitrogen, Carlsbad, CA) for 24 and 48 h, respectively. Metaphase chromosomes for Giemsa *banding* pattern by trypsin digestion with Wright stain (*GTW banding*) were prepared according to standard procedures. Twenty metaphases were karyotyped with GenASIs BandView Analysis System (Applied Spectral Imaging, Carlsbad, CA), and karyograms were described according to the International System for Human Cytogenetic Nomenclature 2016.

### Fluorescent *in-situ* Hybridization (FISH) Assay

FISH analysis on monolayer interphase nuclei and metaphases harvested from bone marrow cultures was undertaken using commercially available FISH probes in the AML panel (Vysis, Abbott Park, IL). Standard FISH hybridization and washing protocols were followed. The slides were then counterstained with the 4′,6-diamidino-2-phenylindole (DAPI). Hybridization signals were captured and analyzed with a GenASIs FISHView Analysis System (Applied Spectral Imaging, Carlsbad, CA). At least 200 cells were scored for each probe set.

### Molecular Pathology Studies

Hematological Malignancy Gene Panel Mutation Analysis, a comprehensive targeted next generation sequencing (NGS) assay, FLT3 internal tandem duplication (ITD) mutation analysis, and JAK2 V617F Mutation Analysis were performed in the Molecular and Genomic Pathology Laboratory of Thomas Jefferson University Hospital. In brief, the NGS panel was designed in-house to detect somatic mutations in 48 genes that are recurrently mutated in myeloid malignancies. Input DNA is processed using the Illumina TruSight(TM) Myeloid Sequencing Panel and sequenced on an Illumina MiSeq sequencer. The assay has sufficient sensitivity to detect mutations present in a heterozygous state at a 5% allele frequency. FLT3 internal tandem duplication (ITD) mutation analysis was performed by amplifying a 329 base-pair fragment of the FLT3 gene including the ITD insertion sites and is sufficient to detect an ITD of 1% allele frequency. The JAK2 V617F mutation analysis was performed using an allelic discrimination assay according to the manufacturer's instruction (MutaScreen Assay, Ipsogen).

## Cases

### Patient #1

#### Clinicopathologic Findings

A 62-year-old female with a history of hypertension and adenocarcinoma of the breast treated with radiation and tamoxifen was referred to our hospital for an abnormal complete blood count (CBC). On admission, her white blood cell (WBC) count was 5 × 10^3^/μL with 17% blasts, hemoglobin was 9.1 g/dL, and platelet count was 22 × 10^3^/μL. Bone marrow biopsy revealed a prominent population of blasts with round to irregular, intermediate sized nuclei, prominent nucleoli, and scant to moderate cytoplasm, comprising 59% by manual count. Several blasts contained dark azurophilic and large salmon colored cytoplasmic granules. Auer rods were not apparent. Concomitant flow cytometry of the bone marrow detected an increased myeloblast population showing the following antigenic profile: CD10–, CD13–, CD14–, CD16–, CD19+ (aberrant), CD33+(dim), CD34+(bright), CD38+, CD56–, CD64–, CD117+, HLA-DR+(bright) (Table 1). This was consistent with a diagnosis of AML.

**Table 1:** Clinicopathologic findings in two patients with variant t(8;21).

**Characteristics**	**Patient #1**	**Patient #2**
Age/Sex	62/F	63/F
Diagnosis	Acute myeloid leukemia	Acute myeloid leukemia
Hemoglobin	9.1 g/dL	9.1 g/dL
Platelet	22,000 B/L	4,000 B/L
WBC	5.0 B/L	6.1 B/L
PB blasts, %	17	67
BM blasts,%	59	75
Cellularity,%	50	60
Morphology	Blasts with round to irregular, intermediate sized nuclei, prominent nucleoli, and scant to moderate cytoplasm; several blasts contained dark azurophilic and large salmon colored cytoplasmic granules; no Auer rods	Medium sized blasts with fine nuclear chromatin, small nucleoli, and scant cytoplasm; no cytoplasmic granules or Auer rods
Immunophenotype	CD10–, CD13–, CD14–, CD16–, CD19+ (aberrant), CD33+(dim), CD34+(bright), CD38+, CD56–, CD64–, CD117+, HLA–DR+(bright), MPO+	CD4+ (dim, partial), CD13– (partial, dim), CD14–, CD33+, CD34+, CD38+ (partial, dim), CD56, CD61 (dim), CD64–, CD117+, and HLA DR+ (dim).

#### Conventional Cytogenetics and FISH

**Figure 1:**
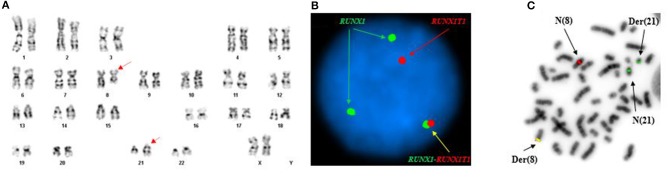
Abnormal cytogenetic and FISH findings in Patient #1. **(A)** Representative karyogram of an apparent t(8;21)(q22;q22) observed in 11 of 20 metaphases analyzed, unable to show submicroscopic small deletions due to cytogenetic technic limitation. The aberration was re-written as der(8)t(8;21)(q22;q22),der(21)del(8)(q22q22)t(8;21) based on FISH findings in **(B,C)**. Arrows indicate aberrant chromosomes. Chromosome numbers are listed on the bottom. **(B)** Interphase FISH study using dual color dual fusion probes demonstrating an atypical pattern with one fusion signal for RUNX1-RUNXT1, one signal (red) for the RUINX1T1 locus, and two signals (green) for the RUNX1T1 locus. **(C)** Metaphase FISH study using dual color dual fusion probes demonstrating a derivative chromosome 8 [der(8)] carrying a fusion signal of RUNX1T1 and RUNX1, a derivative chromosome 21 [der(21)] carrying the green colored RUNX1 signal alone consistent with a sub-microscopic deletion of the rearranged 8q22 segment encompassing the 5′ RUNX1T1, a copy of a normal chromosome 8 [N(8)] and a copy of a normal chromosome 21 [N(21)].

Conventional cytogenetics study revealed an apparently reciprocal translocation between the long arms of chromosomes 8 and 21 at 8q22 and 21q22 [t(8;21)] in twenty of twenty metaphases analyzed. However, FISH on interphase nuclei showed an atypical pattern with one fusion signal representing either der(8) or der(21) (indistinguishable on interphase nuclei) resulting from a typical translocation, two green signals (2G) representing either an intact or partial RUNX1 (AML1) gene at 21q22, and a single red signal (1R) representing either an intact or partial RUNX1T1 (ETO) gene at 8q22. FISH on metaphases showed a derivative chromosome 8 carrying a fusion signal of RUNX1T1 and RUNX1, a derivative chromosome 21 carrying the green colored RUNX1 (AML1) signal alone consistent with an absence of the RUNX1T1 (ETO) signal and indicative of a sub-microscopic deletion following the t(8;21) (the green signal in fact represented the 3′ RUNX1 gene), a copy of a normal chromosome 8 (red), and a copy of a normal chromosome 21 (green) (Figure 1). The final cytogenetic diagnosis for this patient was:

46,XX,der(8)t(8;21)(q22;q22),der(21)del(8)(q22q22)t(8;21)(20).ish der(8)t(8;21)(q22;q22)(3′RUNX1T1+,5′RUNX1+),der(21)del(8)(q22q22)t(8;21)(3′RUNX1+)(3).nuc ish(RUNX1T1x2,RUNX1x3)(RUNX1 con RUNX1T1x1)[165/200], (RPN1,MECOM,EGR1,DEK,D7S522,ASS1,ABL1,CAN,KMT2A,PML,CBFB,RARA,TP53,BCR)x2[200].

#### Molecular Pathology Assays

A pathogenic mutation in the gene RAD21 was identified (Table 2) and the study of internal tandem duplications (ITDs) in the FLT3 gene was negative.

**Table 2:** Hematologic malignancy gene panel findings in two patients with variant t(8;21).

	**Patient #1**			**Patient #2**
**Gene**	**ASXL1**	**DNMT3A**	**IDH1**	**RAD21**
Genomic position (hg19)	chr20:31023092	chr2:25469128	chr2:209113113	chr8:117866547
Nucleotide change	c.2578delA (NM_015338.5)	c.1328_1329dupCT (NM_175629.2)	c.394C>T (NM_005896.2)	c.1097delC (NM_006265.2)
Amino acid change	p.R860Efs^*^7	p.E444Lfs^*^208	p.R132C	p.T366Kfs^*^4
Cosmic ID	None	4678897:4678898	COSM28747	None
Altered allele frequency	44.80%	43.60%	41.30%	41.20%
Classification	Pathogenic	Pathogenic	Pathogenic	Pathogenic

RAD21 encodes a subunit of the cohesin complex, which controls the separation of sister chromatids during mitosis and functions in other processes including transcription and DNA repair. Mutations associated with myeloid malignancies are found throughout the gene and cause inactivation of the protein (16). About 3% of AML and 1–2% of MDS cases are found to have mutations in the RAD21 gene (17, 18). A higher frequency of cohesin defects has been observed in secondary AML and high-risk MDS patients, and cohesin defects have been associated with poor overall survival in MDS patients (16).

#### Outcome

The patient underwent induction with conventional idarubicin and cytarabine. This was complicated only by neutropenic fever without an identified infectious source. A complete cytogenetic remission (CR) was achieved. Molecular profiling was not repeated at the time of her remission bone marrow biopsy. She proceeded immediately to four cycles of consolidation with high-dose cytarabine, which were completed without delays or major complications. She has remained in CR for 18 months.

### Patient #2

#### Clinicopathologic Findings

A 63-year-old female with a history significantly only for a renal mass treated with partial nephrectomy was transferred to our hospital with fatigue, bone pain, and an abnormal CBC. She was found to have a WBC count of 6.1 × 10^3^/μL with 67% blasts, hemoglobin of 9.1 g/dL, and platelets 4 × 10^3^/μL. Bone marrow biopsy showed an increase in number of blasts (75% by manual count). The blasts were medium in size, had fine nuclear chromatin, small nucleoli, and scant cytoplasm. No cytoplasmic granules or Auer rods were seen. Bone marrow flow cytometry detected myeloblasts (69%) with the following antigenic pattern: CD4+ (dim, partial), CD13– (partial, dim), CD14–, CD33+, CD34+, CD38+ (partial, dim), CD56–, CD61–, CD64–, CD117+, and HLA-DR+ (dim) (Table 1). A diagnosis of AML was made.

#### Conventional Cytogenetics and FISH

**Figure 2:**
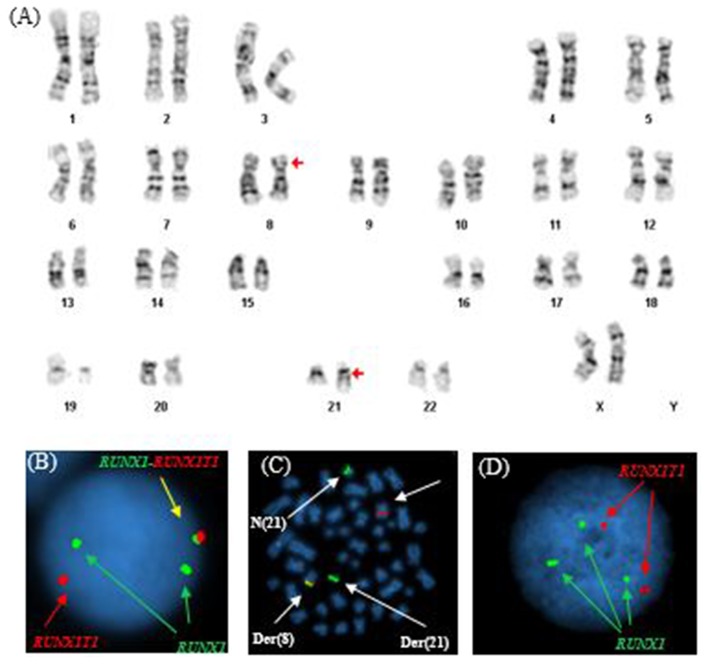
Abnormal cytogenetic and FISH findings in Patient #2. **(A)** Representative karyogram of an apparent t(8;21)(q22;q22) observed in all of 20 metaphases analyzed, unable to show submicroscopic small deletions due to cytogenetic technic limitation. The aberration was re-written as der(8)t(8;21)(q22;q22),der(21)t(8;21)del(8)(q22q22) based on FISH findings in **(B,C)**. Arrows indicate aberrant chromosomes. Chromosome numbers are listed on the bottom. **(B)** Interphase FISH study using dual color dual fusion probes demonstrating an atypical pattern with one fusion signal for RUNX1-RUNXT1, one signal (red) for the RUINX1T1 locus, and two signals (green) for the RUNX1T1 locus. **(C)** Metaphase FISH study using dual color dual fusion probes demonstrating a derivative chromosome 8 [der(8)] carrying a fusion signal of RUNX1T1 and RUNX1, a derivative chromosome 21 [der(21)] carrying the green colored RUNX1 signal alone consistent with a sub-microscopic deletion of the rearranged 8q22 segment encompassing the 5′ RUNX1T1, a copy of a normal chromosome 8 [N(8)] and a copy of a normal chromosome 21 [N(21)]. **(D)** Interphase FISH study using dual color dual fusion probes demonstrating a second atypical pattern with two signals (red) for the RUINX1T1 locus, three signals (green) for the RUNX1T1 locus and no fusion signal for RUNX1-RUNXT1.

Similar to Patient #1, conventional cytogenetics revealed an apparently reciprocal translocation between the long arms of chromosomes 8 and 21 at 8q22 and 21q22 [t(8;21)] in twenty of twenty metaphases analyzed. However, FISH on interphase nuclei showed two atypical signal patterns for the RUNX1/RUNX1T1 translocation probe set: the first was consistent with the presence of a derivative chromosome (one fusion signal) presumably arising from a reciprocal t(8;21) translocation along with one copy of RUNX1T1 (1R) and two copies of RUNX1 (2G), and the second showed two copies of RUNX1T1 (2R) and three copies of RUNX1 (3G). While metaphase FISH analysis later proved that the derivative chromosome represented a der(8), whether the extra RUNX1 signal resulted from a trisomy 21 or from sequential structural alterations following a t(8;21) was unable to be determined. In addition, a low level TP53 deletion was observed (Figure 2). The final cytogenetic diagnosis for this patient was:

46,XX,der(8)t(8;21)(q22;q22),der(21)t(8;21)del(8)(q22q22)(20).nuc ish(RUNX1T1x2,RUNX1x3)(RUNX1 conRUNX1T1x1)[92/200]/(RUNX1T1x2,RUNX1x3)[92/200], (TP53x1,CEP17x2)[37/200], (ASS1,ABL1,PML,CBFB,RARA,BCR)x2[200].

#### Molecular Pathology Assays

Pathogenic mutations were found in three genes: ASXL1, DNMT3A, and IDH1 (Table 2). The studies of JAK2 V617F mutation and internal tandem duplications (ITDs) in the FLT3 gene were both negative.

ASXL1 encodes a chromatin-binding Polycomb group (PcG) protein involved in transcriptional regulation (19). Somatic mutations in ASXL1 have been reported in 10.8% adults with *de novo* AML, and in 17.2% of AML cases with intermediate risk cytogenetics (20, 21). DNMT3A encodes DNA (cytosine-5)-methyltransferase 3A and is essential for establishing genome-wide patterns of CpG methylation during development. It is also important for regulating gene expression, parental imprinting, and maintaining genome integrity (22). The Cancer Genome Atlas Research Network identified mutations in the DNMT3A gene in 51/200 (26%) *de novo* AML samples (17). Isocitrate dehydrogenases, which include IDH1 and IDH2, catalyze the oxidative decarboxylation of isocitrate to 2-oxoglutarate (alpha-ketoglutarate) (23, 24). Approximately 6–9% of AML cases are found to have mutations in the IDH1 gene, with a higher frequency in normal karyotype-AML (8–16%) (19). Studies of the prognostic significance of ASXL1, DNMT3A, and IDH mutations in AML have reported complex and sometimes conflicting results, although most reports support negative effects on prognosis (19–21). The co-occurrence of mutations in epigenetic regulators, including ASXL1, DNMT3A, and IDH1, has also been reported (25–27). However, the frequency and clinical consequences of this combination of mutations in core binding factor AML are not well-characterized.

#### Outcome

The patient underwent treatment with conventional idarubicin and cytarabine induction. This was complicated by neutropenic fever without an identified infectious source and the development of a rash that was thought to be due to cytarabine. A cytogenetic CR was achieved. Molecular profiling was not repeated at the time of her remission bone marrow biopsy. She proceeded immediately to consolidation with high dose cytarabine. Unfortunately, she completed 3 cycles and then her disease relapsed. Cytogenetics at the time of relapse showed the same t(8;21), and molecular profiling identified the same mutations in ASXL1, DNMT3A, and IDH1 that were present at diagnosis. She underwent re-induction on a clinical trial, however, her disease was refractory. She subsequently began treatment with an IDH1 inhibitor and her disease was stable for ~4 months. At the time of disease progression, she opted for hospice. The patient died 14 months after her diagnosis.

## Discussion

Although AML with t(8;21)(q22;q22) is generally is associated with a favorable prognosis, it is unclear if the same can be said for variant t(8;21) (1–3, 11). Our patients had similar variant subtypes of t(8;21) and were treated with idarubicin and cytarabine induction therapy. Both achieved a complete remission, however, Patient #1 has remained in CR after consolidation chemotherapy, while Patient #2 relapsed and subsequently died.

Published outcomes for other variant cases are similarly heterogeneous. Kawakami et al. (13) described a case of a 37-year-old man with a variant t(8;21) that demonstrated a three way translocation between chromosomes 8,9, and 21. This patient had an AML1/ETO fusion transcript that was identical to the fusion transcript found in patients with classic t(8;21), however, he did not achieve a complete remission with idarubicin and cytarabine (13). Similarly, a 15-year-old patient with a four way translocation t(8;17;15;21)(q22;q23;q15;q22) was reported, and he also showed rapid progression of his disease (28). An additional variant form of t(8;21) was reported in a 10-year-old female with a translocation between chromosomes 4, 8, and 21, with loss of the X chromosome and a gain of chromosome 6. She had an early relapse and a poor outcome (29).

Another paper reported three cases of variant t(8;21) AML. Two patients had three way translocations; the first with a translocation between chromosomes 8, 18, and 21 as well as a del(7)(q32q34) and the second with a translocation involving chromosomes 2, 8, and 21 with loss of the Y chromosome. The third patient had a derivative eight with the interstitial inverted insertion of 21q and concurrent monosomy 21; this patient achieved CR for 15 months before relapse (11). Another author presented four patients with variant forms of t(8;21). Two of these patients had three-way translocations, one with t(1;8;21) and the other with t(8;11;21), and a third patient had a four way translocation between chromosomes 4, 8, 12, and 21. The fourth patient reported had three neoplastic clones in which the segment of chromosome 8 containing bands q22 through q24.1 had been duplicated and inverted (the t(8;21) had been inserted within the duplicated segment. All four of these patients achieved a CR (30). Another patient described in the literature with a complex translocation involving chromosomes 1, 8, and 21 with del 9q22 and loss of the X chromosome had no reported outcome (31).

Although the prognosis for patients with variant t(8;21) is generally unclear, certain markers have been associated with outcomes. For example, loss of the sex chromosome and del (9q) have been associated with shorter overall survival, and trisomy 4 has been thought to be associated with a poorer prognosis (11, 13). AML1/ETO frequently expresses positivity for CD19 and CD56 and, although CD19 positivity is not thought to alter prognosis, it has been reported that CD56 is associated with shorter remission time and survival. Additionally, c-kit and EML are thought to be associated with a negative outcome (13).

The cytogenetic abnormalities that occur from t(8;21)(q22;q22) result in the disruption of a transcription factor that functions as a regulator of hematopoiesis. In patients with classical t(8;21) AML, remission rates after induction chemotherapy with an anthracycline and cytarabine approaches 90% (10). Although these responses are considered favorable, the median survival for these patients, according to long term follow up reports, is 5 years or less (2). Variant t(8;21) is much less common than traditional CBF AML, and it has been reported that the majority of patients with variant rearrangements have a complex translocation involving a third chromosome (28). The reported responses to therapy for these patients are varied.

Little is known about the molecular landscape of patients with variant t(8;21). Previously reported cases did not include molecular data, likely because many were published prior to the routine use of NGS in AML diagnosis. However, extrapolation from the body of literature regarding molecular mutations in classical t(8;21) AML may be possible. In general, tyrosine kinase mutations such as KIT and FLT3 are most common in this subset of patients and co-mutations in these genes can be seen (32–34). Mutations in KIT have been clearly shown to correlate with shortened remission duration and decreased overall survival in t(8;21) AML (35, 36). The prognostic significance of KIT mutations in inv(16) AML is less clearly defined (37, 38). Mutations in epigenetic regulators and members of the cohesin complex, including ASXL1, IDH1/2, and RAD21, have also been identified in a significant subset of t(8;21) AML. Interestingly, these mutations are vanishingly rare in inv(16) AML (32). Whether the genetic profile of variant t(8;21) is similar to that of classical t(8;21) remains to be determined, however, the patients presented here do share similar molecular signatures to those described in CBF AML.

We provide the first description of two cases of variant t(8;21) along with their molecular profiles. Additional reports of similar cases are needed in order to better determine the interplay between, and the clinical significance of, the cytogenetic and molecular abnormalities. The two patients presented in this paper with variant t(8;21) both achieved complete remission with standard induction therapy, however, only one had a durable response.

## Data Availability Statement

The raw data supporting the conclusions of this manuscript will be made available by the authors, without undue reservation, to any qualified researcher.

## Ethics Statement

Written informed consent was obtained from the individual(s) for the publication of any potentially identifiable images or data included in this article.

## Author Contributions

LW, JC, Z-XW, and JL provided substantial contribution to the conception, drafting, editing, and final approval of this manuscript.

### Conflict of Interest

The authors declare that the research was conducted in the absence of any commercial or financial relationships that could be construed as a potential conflict of interest. The handling Editor declared a shared affiliation, though no other collaboration, with one of the author LW.
